# Close Correlation between Frailty and Depressive State in Chronic Liver Diseases

**DOI:** 10.3390/medicina56070319

**Published:** 2020-06-27

**Authors:** Hiroki Nishikawa, Kazunori Yoh, Hirayuki Enomoto, Yoshinori Iwata, Yoshiyuki Sakai, Kyohei Kishino, Yoshihiro Shimono, Naoto Ikeda, Tomoyuki Takashima, Nobuhiro Aizawa, Ryo Takata, Kunihiro Hasegawa, Takashi Koriyama, Yukihisa Yuri, Takashi Nishimura, Shuhei Nishiguchi, Hiroko Iijima

**Affiliations:** 1 Department of Internal Medicine, Division of Gastroenterology and Hepatology, Hyogo College of Medicine, Nishinomiya, Hyogo 663-8501, Japan; mm2wintwin@ybb.ne.jp (K.Y.); enomoto@hyo-med.ac.jp (H.E.); yo-iwata@hyo-med.ac.jp (Y.I.); sakai429@hyo-med.ac.jp (Y.S.); hcm.kyohei@gmail.com (K.K.); yoshihiro19870729@yahoo.co.jp (Y.S.); nikeneko@hyo-med.ac.jp (N.I.); tomo0204@yahoo.co.jp (T.T.); nobu23hiro@yahoo.co.jp (N.A.); chano_chano_rt@yahoo.co.jp (R.T.); hiro.red1230@gmail.com (K.H.); takashi051114@yahoo.co.jp (T.K.); gyma27ijo04td@gmail.com (Y.Y.); tk-nishimura@hyo-med.ac.jp (T.N.); hiroko-i@hyo-med.ac.jp (H.I.); 2 Center for Clinical Research and Education, Hyogo College of Medicine, Nishinomiya, Hyogo 663-8501, Japan; 3 Kano General Hospital, Osaka, Osaka 531-0041, Japan; nishiguchi@heartfull.or.jp

**Keywords:** chronic liver disease, frailty, depression, physiological aspects, psychological aspects

## Abstract

Background and objectives: Few data with regard to the relevance between depression and frailty in chronic liver disease (CLD) patients are currently available. We aimed to elucidate the relationship between frailty and depression as evaluated by the Beck Depression Inventory—2nd edition (BDI-II) in CLD patients (*n* = 340, median age = 65.0 years). Methods: Frailty was defined as a clinical syndrome in which three or more of the following criteria were met: body weight loss, exhaustion, muscle weakness, slow walking speed and low physical activity. Depressive state was defined as BDI-II score 11 or greater. Results: Robust (frailty score = zero), prefrail (frailty score = one or two) and frailty were identified in 114 (33.5%), 182 (53.5%) and 44 (12.9%). The median BDI-II score was five. Depressive state was identified in 84 patients (24.7%). The median BDI-II scores in patients with robust, prefrail and frail traits were 2, 7 and 12.5 (robust vs. prefrail, *p* < 0.0001; prefrail vs. robust, *p* = 0.0003; robust vs. frail, *p* < 0.0001; overall *p* < 0.0001). The proportions of depressive state in patients with robust, prefrail and frail traits were 3.51%, 30.77% and 54.55% (robust vs. prefrail, *p* < 0.0001; prefrail vs. robust, *p* = 0.0046; robust vs. frail, *p* < 0.0001; overall *p* < 0.0001). BDI-II score significantly correlated with frailty score (*r_s_* = 0.5855, *p* < 0.0001). Conclusions: The close correlation between frailty and depression can be found in CLD. Preventing frailty in CLD should be approached both physiologically and psychologically.

## 1. Introduction

Frailty refers to a condition in which the physical and mental vitality (motor function, cognitive function, etc.) declines with aging [1,2,3]. Since Japan is an aging society, interest in frailty is high. Acknowledgement of the condition and signs of frailty makes it easier to predict and prevent subsequent physical, psychological and social health problems [1,2,3]. Frailty can be prevented by appropriate interventions [1,2,3].

The disease concept of frailty has been recently extended to the field of chronic liver diseases (CLDs) as clinical manifestations of poor global physical function [4,5,6,7,8,9]. In patients with decreased liver functional reserve, frailty can develop independent of age due to liver disease specific protein–energy disorders or other metabolic orders, which can be a point for debate among clinicians [4,5,6,7,8,9]. Cognitive and physical frailty are common in patients with liver cirrhosis (LC) [10].

Depressive state is often encountered in patients with CLDs [11,12,13,14,15,16,17,18]. Even in CLD patients with an earlier liver fibrosis stage, depressive manifestations can be frequently found compared with healthy individuals [17]. In patients with chronic hepatitis C, depressive state, anxiety, and fatigue were demonstrated to be major psychiatric abnormalities [19]. In patients with nonalcoholic fatty liver disease, risk ratio for the development of depression is reported to increase according to the disease severity [20]. Poor quality of life and increasing medical costs have been reported for CLD patients with depressive state [14,21]. In patients with LC, depressive state can be linked to substantial morbidity and mortality. Nevertheless, it is often overlooked and is not given full consideration in the current practice guidelines [11]. In general, emotional well-being and mental health in patients with chronic diseases are often under-recognized and untreated by healthcare workers. Depressive state seems to be one of essential neurocognitive manifestations in CLD patients [11]. Interventions for CLD patients with depression may be beneficial in order to improve health related quality of life. The Beck Depression Inventory—2nd edition (BDI-II) is one of most widely used and well validated screening questionnaires for depression [22].

A previous report demonstrated that depression as assessed by the 15-question geriatric depression scale is common in patients with end stage liver disease and is strongly associated with frailty [23]. Another report demonstrates that in LC outpatients 18–80 years of age, patients with depression as assessed by the mini international neuropsychiatric interview had higher frailty scores [11]. However, as far as we know, few data with regard to the relevance between depression and frailty in CLD patients (including both LC patients and non-LC patients) are currently available. In this study, we sought to elucidate the relationship between frailty and depression as evaluated by BDI-II in CLD patients.

## 2. Patients and Methods

### 2.1. Patients

In our hospital, anthropometric measurements, muscle strength measurements and medical interview were completed with the agreement of patients. A total of 340 CLD patients with data for frailty and the BDI-II score evaluable who admitted to our hospital between July 2015 and March 2020 were analyzed. LC was decided as described elsewhere [24]. Frailty was defined as a clinical syndrome in which 3 or more of the following criteria were met (frailty score 3, 4 or 5): unintentional body weight (BW) loss (2, 3 kg or more BW loss within the past 6 months), self-reported exhaustion, muscle weakness (grip strength (GS): <26 kg in men and <18 kg in women), slow walking speed (WS, <1.0 m/s) and low physical activity (doing light exercise or not), while prefrail was defined as patients with one or two above mentioned phenotype (frailty score 1 or 2). Patients with none of 5 phenotypes were considered as having robust status (frailty score 0) [25,26]. These criteria are presented by Satake et al. as Japanese version of cardiovascular health study (CHS) criteria (J-CHS criteria) [25]. GS was tested based on the current guidelines [27]. In all analyzed subjects, a 6-meter walking test was done. A 6-meter walking test was done twice with all subjects; the WS (m/s) was defined as the mean value of them.

### 2.2. Questionnaire Using BDI-II

The BDI-II is a globally used screening questionnaire for the severity of depression [28,29]. The BDI-II has favorable psychometric properties, high reliability and internal consistency and is a self-reported questionnaire that comprises 21 items, and each answer is evaluated on a four-point scale (i.e., 0 to 3 points) [28,30]. Higher BDI-II score indicates a severer depressive state. Patients were classified into the following groups: normal (BDI-II score = 0–10), and the severity of depression as minimal (BDI-II score=11–16), mild (BDI-II score = 17–20), moderate (BDI-II score = 21–30) and severe (BDI-II score, 31 or greater) [29,31,32,33]. In this study, patients with depressive state were defined as those with BDI-II score 11 or greater. Patients with overt hepatic encephalopathy and/or large ascites were excluded due to lacking in reliability for self-reported questionnaire.

### 2.3. Our Study

We retrospectively examined the relationship between the frailty status and BDI-II score. The ethical approval was obtained from the ethics committee of our hospital (approval no. 3469, date of approval: 27 March 2020). The protocol in the study rigorously observed all regulations of the Declaration of Helsinki.

### 2.4. Statistical Considerations

The JMP 14 software (SAS Institute, Inc., Cary, NC, USA) was used to analyze statistically. For the continuous variables, those with normal distribution were compared by Student’s *t*-test (2-group comparison) or analysis of variance (3-group comparison), and those without normal distribution were compared by Mann–Whitney *U*-test (2-group comparison) or Kruskal–Wallis test (3-group comparison). In terms of the correlation between two parameters, Spearman’s rank coefficient *r_s_* was employed. Continuous variables were demonstrated as medians and interquartile ranges (IQRs) in parenthesis. Categorical variables were demonstrated as the number of patients and percentages in parentheses. The chi-squared test was used to evaluate the group differences in categorical variables. Significant parameters correlated with frailty score were entered into the multivariate regression analysis with multiple predictive parameters by least squares method to identify candidates. A level of *p* = 0.05 was used to denote statistical significance.

## 3. Results

### 3.1. Patient Data

This was a retrospective cross-sectional study. Table 1 summarizes the data in this study. LC was identified at baseline in 121 cases (35.6%). There were 255 patients (75.0%) with albumin–bilirubin (ALBI) grade 1, 76 (22.4%) with ALBI grade 2 and 9 (2.6%) with ALBI grade 3 [34]. Forty-six patients (13.5%) had the WS decrease (i.e., <1.0 m/s). Twenty-six patients (16.9%) in male and 49 patients (26.3%) in female had the GS decrease (i.e., <26 kg in male and <18 kg in female). One hundred and sixty-eight patients (49.4%) reported exhaustion. Twelve patients (3.5%) reported BW loss. Eighty-three patients (24.4%) reported low physical activity. Frailty score ranged from 0 to 5 (median, 1). Robust (frailty score 0), prefrail (frailty score 1 or 2) and frailty (frailty score 3 or more) were identified in 114 (33.5%), 182 (53.5%) and 44 (12.9%), respectively. Depressive state (BDI-II score 11 or greater) was identified in 84 patients (24.7%). There were 256 patients (75.3%) with normal state, 44 patients (12.9%) with minimal depressive state, 20 (5.9%) with mild depressive state, 10 patients (2.9%) with moderate depressive state and 10 (2.9%) with severe depressive state. In 21 items of BDI-II questionnaire, the question with the highest prevalence of 3 point (the worst point) was the question about sexual desire (107 patients (31.5%) had no sexual desire).

### 3.2. BDI-II Score among GROUPS of Robust, Prefrail and Frailty for All Cases

**Figure 1:**
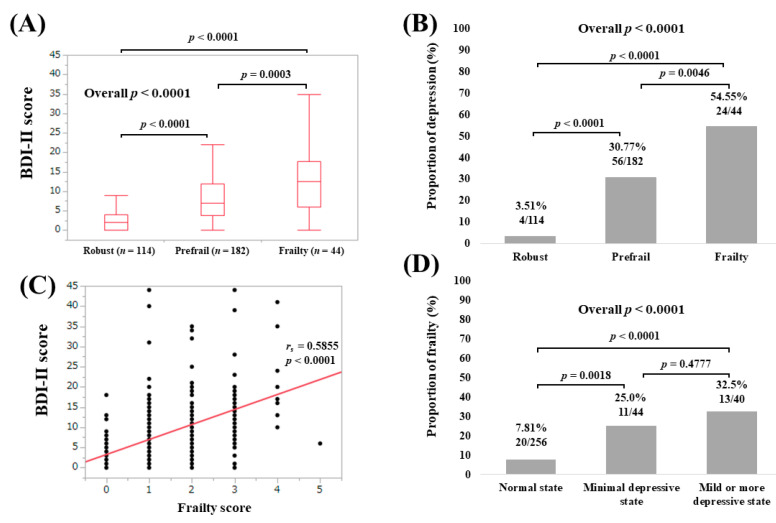
(**A**) BDI-II score among groups of robust, prefrail and frailty for all cases; (**B**) proportion of depressive state (BDI-II score, 11 or greater) among groups of robust, prefrail and frailty for all cases; (**C**) correlation between BDI-II score and frailty score for all cases; (**D**) proportion of frailty according to the severity of depressive state. BDI-II; Beck Depression Inventory—2nd edition.

The median (IQR) BDI-II scores in patients with robust (*n* = 114), prefrail (*n* = 182) and frailty (*n* = 44) were 2 (0, 4), 7 (3.75, 12) and 12.5 (6, 17.75), respectively (overall *p* < 0.0001; Figure 1A). The proportions of depressive state in patients with robust, prefrail and frailty were well stratified (overall *p* < 0.0001; Figure 1B). The BDI-II score significantly correlated with frailty score (*r_s_* = 0.5855, *p* < 0.0001; Figure 1C) The proportion of frailty in patients with normal state, minimal depressive state and mild or greater depressive state were well stratified (overall *p* value < 0.0001; Figure 1D).

### 3.3. The Proportion of Depressive State According to the 5 Phenotypes of Frailty

**Figure 2:**
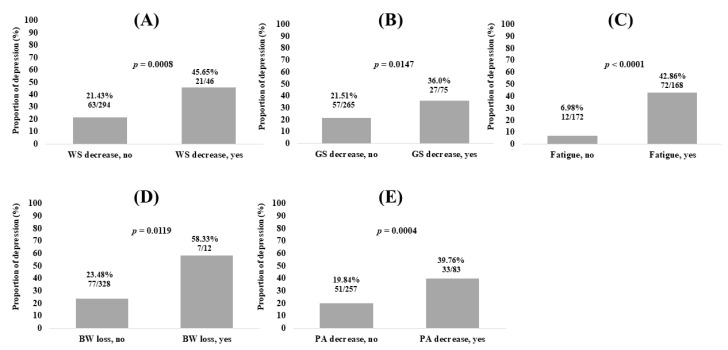
Proportion of depressive state (BDI-II score, 11 or greater) according to frailty phenotype. (**A**) walking speed; (**B**) grip strength; (**C**) Fatigue; (**D**) body weight loss; (**E**) physical activity (PA).

Patients with WS decrease had significantly higher prevalence of depressive state than those without (*p* = 0.0008; Figure 2A). Patients with GS decrease had significantly higher proportion of depressive state than those without (*p* = 0.0147; Figure 2B). Patients with fatigue had significantly higher prevalence of depressive state than those without (*p* < 0.0001; Figure 2C). Patients with BW loss had significantly higher proportion of depressive state than those without (*p* = 0.0119; Figure 2D). Patients with low physical activity had significantly higher prevalence of depressive state than those without (*p* = 0.0004; Figure 2E). 

### 3.4. Subset Analysis 1: BDI-II Score among Groups of Robust, Prefrail and Frailty in LC Patients

**Figure 3:**
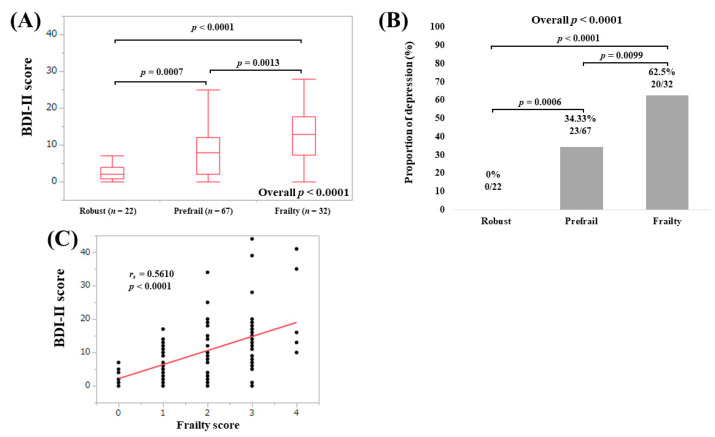
(**A**) BDI-II score among groups of robust, prefrail and frailty in LC patients; (**B**) proportion of depressive state (BDI-II score, 11 or greater) among groups of robust, prefrail and frailty in LC patients; (**C**) correlation between BDI-II score and frailty score in LC patients.

The median (IQR) BDI-II scores in LC patients with robust (*n* = 22), prefrail (*n* = 67) and frailty (*n* = 32) were 2 (0.75, 4), 8 (2, 12) and 13 (7.25, 17.75), respectively (overall *p* < 0.0001; Figure 3A). The proportions of depressive state in LC patients with robust, prefrail and frailty were well stratified (overall *p* < 0.0001; Figure 3B). BDI-II score significantly correlated with frailty score (*r_s_* = 0.5610, *p* < 0.0001; Figure 3C).

### 3.5. Subset Analysis 2: BDI-II Score among Groups of Robust, Prefrail and Frailty in Non-LC Patients

**Figure 4:**
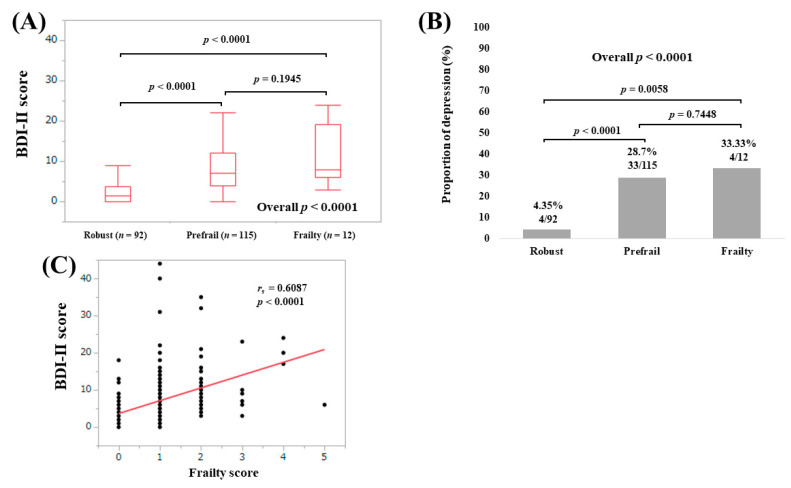
(**A**) BDI-II score among groups of robust, prefrail and frailty in non-LC patients; (**B**) proportion of depressive state (BDI-II score, 11 or greater) among groups of robust, prefrail and frailty in non-LC patients; (**C**) correlation between BDI-II score and frailty score in non-LC patients.

The median (IQR) BDI-II scores in non-LC patients with robust (*n* = 92), prefrail (*n* = 115) and frailty (*n* = 12) were 1.5 (0, 3.75), 7 (4, 12) and 8 (6, 19.25), respectively (overall *p* < 0.0001; Figure 4A). The proportions of depressive state in non-LC patients with robust, prefrail and frailty were well stratified (overall *p* < 0.0001; Figure 4B). BDI-II score significantly correlated with frailty score (*r_s_* = 0.6087, *p* < 0.0001) (Figure 4C).

### 3.6. Subset Analysis 3: BDI-II Score among Groups of Robust, Prefrail and Frailty in Elderly Patients (65 Years or More)

**Figure 5:**
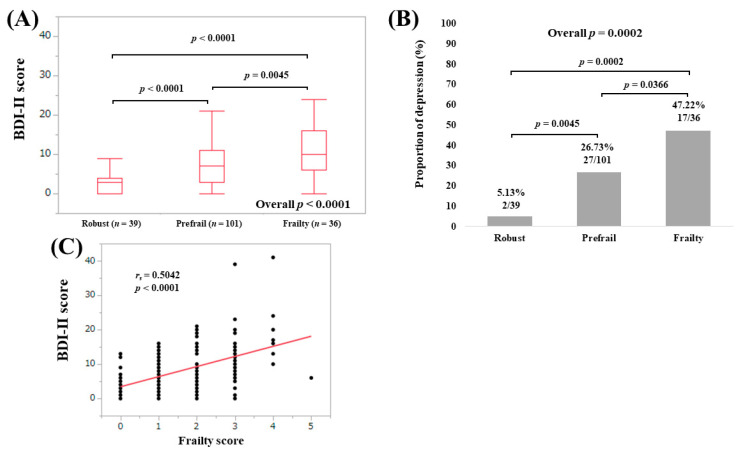
(**A**) BDI-II score among groups of robust, prefrail and frailty in patients aged 65 years or older; (**B**) proportion of depressive state (BDI-II score, 11 or greater) among groups of robust, prefrail and frailty in patients aged 65 years or older; (**C**) correlation between BDI-II score and frailty score n patients aged 65 years or older.

The median (IQR) BDI-II scores in elderly patients (65 years or more) with robust (*n* = 39), prefrail (*n* = 101) and frailty (*n* = 36) were 3 (0, 4), 7 (3, 11) and 10 (6, 16), respectively (overall *p* < 0.0001; Figure 5A). The proportions of depressive state in patients aged 65 years or older with robust, prefrail and frailty were well stratified (overall *p* = 0.0002; Figure 5B). BDI-II score significantly correlated with frailty score (*r_s_* = 0.5042, *p* < 0.0001; Figure 5C).

### 3.7. Subset Analysis 4: BDI-II Score among Groups of Robust, Prefrail and Frailty in Elderly Patients (Less Than 65 Years)

**Figure 6:**
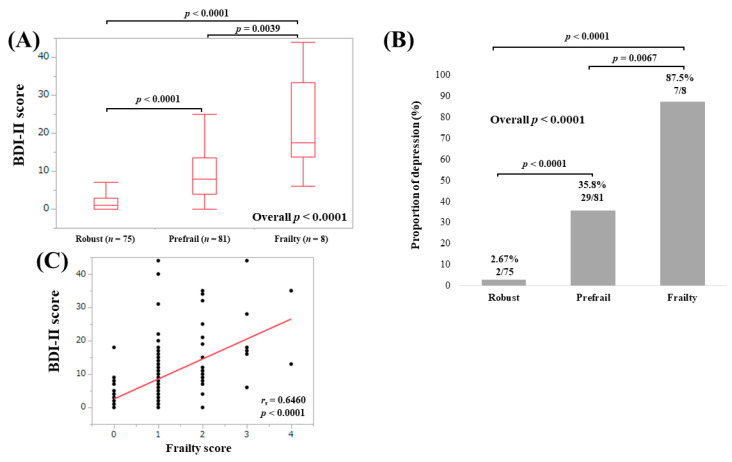
(**A**) BDI-II score among groups of robust, prefrail and frailty in patients less than 65 years; (**B**) proportion of depressive state (BDI-II score, 11 or greater) among groups of robust, prefrail and frailty in patients less than 65 years; (**C**) correlation between BDI-II score and frailty score in patients less than 65 years. BDI-II; Beck Depression Inventory—2nd edition.

The median (IQR) BDI-II scores in patients less than 65 years with robust (*n* = 75), prefrail (*n* = 81) and frailty (*n* = 8) were 1 (0, 3), 8 (4, 13.5) and 17.5 (13.75, 33.25), respectively (overall *p* < 0.0001; Figure 6A). The proportions of depressive state in patients less than 65 years with robust, prefrail and frailty were well stratified (overall *p* < 0.0001; Figure 6B). BDI-II score significantly correlated with frailty score (*r_s_* = 0.6460, *p* < 0.0001; Figure 6C).

### 3.8. Univariate and Multivariate Analyses of Continuous Parameters Linked to The Frailty Score

Correlation coefficients between frailty score (0–5) and continuous parameters for all cases were presented in Table 2. Significant factors were: age (*r_s_* = 0.3312, *p* < 0.0001), serum albumin (*r_s_* = −0.2888, *p* < 0.0001), prothrombin time (*r_s_* = −0.1697, *p* = 0.0017), platelet count (*r_s_* = −0.1829, *p* = 0.0007), aspartate aminotransferase (*r_s_* = 0.1307, *p* = 0.0159), total cholesterol (*r_s_* = −0.1255, *p* = 0.0208) and BDI-II score (*r_s_* = 0.5855, *p* < 0.0001). In the multivariate analyses of factors associated with the frailty score, age (*p* < 0.0001), serum albumin (*p* = 0.0008) and BDI-II score (*p* < 0.0001) were observed to be significant (Table 2).

## 4. Discussion

The concept of overlap in frailty and depression is not so amazing provided that the frailty phenotype and depression share the criteria of exhaustion from a measure of depression at the outset [35,36]. In our previous report, we demonstrated that sarcopenia as evaluated by muscle muss decline and muscle strength decline in CLDs was closely associated with depression [24]. However, relevant data between depression and frailty in CLD patients are currently scarce. To clarify the relationship between frailty and depression in CLDs appears to be clinically meaningful. In our results, BDI-II score was well stratified according to the frailty status for all cases and all subgroups. Correlation coefficients between frailty score and BDI-II score were over 0.5 for all analyses, and notably that in patients less than 65 years was 0.6460. Multivariate analysis revealed that BDI-II score was an independent factor linked to the frailty score. These results indicated that close correlation between frailty and depressive state exactly exists in patients with CLDs. The screening of psychological distress characterized by depressive state should be strengthened in CLD patients. In our country, medical check-up for frailty has been launched in patients aged 75 years or older. This aims to identify patients with frailty or prefrail in the early phase and to give them appropriate interventions. In general, elderly patients may have several comorbid diseases. In that sense, our current data may be clinically of importance. In the field of CLDs, target population in previous reports with regard to frailty is limited to LC patients [11,23]. The major strength of our study is that the close relationship between frailty and depressive state was shown not only in LC patients, but also in non-LC patients. In our data, similar results were obtained in non-LC patients compared with LC patients. Reviewing our results, the close correlation between frailty and depression can be extended to non-LC patients as well as LC patients.

A recent meta-analysis reported that the overall prevalence of depression in 8023 elderly people with frailty was 38.6% [37]. While in our data, out of 44 patients with frailty, 24 patients (54.55%) had depressive state, which was higher than their data. This may be due to the long-standing disease burden of CLD itself. Many CLD patients have a long history of disease.

In our previous study, we stated that reasons for the high prevalence of CLD patients with depressive state include the long-term suffering caused by CLD itself, social pressure for working and economic pressure for medical therapies [24]. On the other hand, the proportion of depressive state in prefrail patients accounted for 30.77% in our data. In patients with depressive state and prefrail (*n* = 56), 49 patients (87.5%) reported exhaustion. The question about exhaustion in daily clinical practice in CLD patients appears to be pivotal due to its close linkage to frailty or depressive state.

In our data, out of eight patients with frailty aged less than 65 years, seven had depressive state. Of these, six (85.7%) had LC. In the previous report, a stepwise increment in depressive state score by frailty score was shown, and compared with non-frail patients, the frailty patients had higher model for end stage liver disease scores, lower serum sodium and albumin levels and higher prevalence of hepatic encephalopathy [23]. In addition, the mean (standard deviation) age in patients with frailty in their study was 55.8 (9.2) [23]. This means that most their frailty patients was less than 65 years. Frailty assessment in the clinical settings should not be restricted to elderly patients. Furthermore, in our multivariate analysis, serum albumin revealed to be a significant factor associated with the frailty score as well as age and BDI-II score. Disease specific frailty condition should be emphasized. In the current guidelines for sarcopenia in liver diseases proposed by Japan society of hepatology, age restriction is omitted because some younger CLD patients can involve sarcopenia [27].

Several limitations to our study are necessary to be acknowledged. First, this study was a single-center cross-sectional observational study with a retrospective nature. Second, the study data were derived from a Japanese CLD population data, and additional exams on ethnically diverse populations are necessary to further verify and extend the application to ethnically diverse populations. Third, GS or WS values (i.e., one of phenotypes for frailty measurement) can change depending on measurement conditions. Fourth, patients with overt hepatic encephalopathy and/or large ascites who are potentially involved in frailty were excluded due to lacking in reliability for self-reported questionnaire, creating bias. Fifth, due to the cross-sectional study design of this study, whether frailty caused depressive state or vice versa was unclear. Our data should be therefore interpreted carefully. Nevertheless, our results suggested that frailty in Japanese CLD patients can be closely linked to depressive state.

## 5. Conclusions

The close correlation between frailty and depression can be found in CLD patients, and these tendencies may be identified even in prefrail patients. Preventing frailty in patients with CLDs should be approached from both physiological and psychological aspects.

## Figures and Tables

**Table 1:** Baseline characteristics (*n* = 340).

Variables	All Cases (*n* = 340)
Age (years)	65 (54, 72)
Gender, male/female	154/186
Liver disease etiology HCV associated/HBV associated/others	172/59/109
Presence of frailty, yes/no	44/296
Presence of LC, yes/no	121/219
	
Body mass index (kg/m^2^)	22.9 (20.5, 25.9)
Walking speed (m/s)	1.31 (1.13, 1.46)
Grip strength (kg), male	33.7 (28.4, 39.8)
Grip strength (kg), female	21.0 (17.7, 24.4)
Total bilirubin (mg/dL)	0.8 (0.6, 1.1)
Serum albumin (g/dL)	4.3 (3.925, 4.5)
ALBI score	−2.9 (−3.12, −2.6)
ALBI grade, 1/2/3	255/76/9
Prothrombin time (%)	91.2 (80.5, 98.7)
Platelet count (× 10^4^/mm^3^)	17.9 (12.5, 22.3)
AST (IU/L)	25 (19.3, 33.0)
ALT (IU/L)	19 (14, 32)
Total cholesterol (mg/dL)	183 (154, 215)
BDI-II score	5 (2, 10)

Data expressed as number or median value (interquartile range). HCV; hepatitis C virus, HBV; hepatitis B virus, LC; liver cirrhosis, ALBI; albumin–bilirubin, AST; aspartate aminotransferase, ALT; alanine aminotransferase, BDI-II; Beck Depression Inventory—2nd edition.

**Table 2:** Univariate and multivariate analyses of continuous parameters associated with the frailty score.

	Univariate Analysis		Multivariate Analysis		
	*r_s_*	*p*-Value	Estimates	SE	*p*-Value
Age	0.3312	<0.0001	0.024	0.004	<0.0001
BMI	−0.0755	0.1539			
Total bilirubin	0.0531	0.3285			
Serum albumin	−0.2888	<0.0001	−0.353	0.105	0.0008
Prothrombin time	−0.1697	0.0017	−0.0006	0.001	0.5082
Platelet count	−0.1829	0.0007	0.001	0.008	0.8900
AST	0.1307	0.0159	0.0025	0.0024	0.2964
ALT	0.0108	0.8421			
Total cholesterol	−0.1255	0.0208	0.0005	0.0012	0.6676
BDI-II score	0.5855	<0.0001	0.066	0.006	<0.0001

SE; standard error, BMI; body mass index, AST; aspartate aminotransferase, ALT; alanine aminotransferase, BDI-II; Beck Depression Inventory—2nd edition.

## Abbreviations

CLD: chronic liver disease, LC; liver cirrhosis, BDI-II; Beck Depression Inventory—2nd edition, BW; body weight, GS; grip strength, WS; walking speed, J-CHS; Japanese version of cardiovascular health study, IQR; interquartile range, ALBI; albumin–bilirubin.
